# Predicting mutually exclusive spliced exons based on exon length, splice site and reading frame conservation, and exon sequence homology

**DOI:** 10.1186/1471-2105-12-270

**Published:** 2011-06-30

**Authors:** Holger Pillmann, Klas Hatje, Florian Odronitz, Björn Hammesfahr, Martin Kollmar

**Affiliations:** 1 Abteilung NMR basierte Strukturbiologie, Max-Planck-Institut für Biophysikalische Chemie, Am Fassberg 11, D-37077 Göttingen, Germany

## Abstract

**Background:**

Alternative splicing of pre-mature RNA is an important process eukaryotes utilize to increase their repertoire of different protein products. Several types of different alternative splice forms exist including exon skipping, differential splicing of exons at their 3'- or 5'-end, intron retention, and mutually exclusive splicing. The latter term is used for clusters of internal exons that are spliced in a mutually exclusive manner.

**Results:**

We have implemented an extension to the WebScipio software to search for mutually exclusive exons. Here, the search is based on the precondition that mutually exclusive exons encode regions of the same structural part of the protein product. This precondition provides restrictions to the search for candidate exons concerning their length, splice site conservation and reading frame preservation, and overall homology. Mutually exclusive exons that are not homologous and not of about the same length will not be found. Using the new algorithm, mutually exclusive exons in several example genes, a dynein heavy chain, a muscle myosin heavy chain, and Dscam were correctly identified. In addition, the algorithm was applied to the whole *Drosophila melanogaster *X chromosome and the results were compared to the Flybase annotation and an *ab initio *prediction. Clusters of mutually exclusive exons might be subsequent to each other and might encode dozens of exons.

**Conclusions:**

This is the first implementation of an automatic search for mutually exclusive exons in eukaryotes. Exons are predicted and reconstructed in the same run providing the complete gene structure for the protein query of interest. WebScipio offers high quality gene structure figures with the clusters of mutually exclusive exons colour-coded, and several analysis tools for further manual inspection. The genome scale analysis of all genes of the *Drosophila melanogaster *X chromosome showed that WebScipio is able to find all but two of the 28 annotated mutually exclusive spliced exons and predicts 39 new candidate exons. Thus, WebScipio should be able to identify mutually exclusive spliced exons in any query sequence from any species with a very high probability. WebScipio is freely available to academics at http://www.webscipio.org.

## Background

Eukaryotes can enhance their repertoire of different protein products by alternative splicing of the corresponding genes [1]. Since the first description of alternative splicing of precursor mRNA almost 30 years ago [2,3] the suggested and verified percentage of human genes that are spliced into alternative transcripts has steadily risen (for reviews see for example [4,5]). Very recently, two studies using high-throughput sequencing indicate that every single human gene containing more than one exon is transcribed and processed to yield multiple mRNAs [6,7].

Mainly, five different types of alternative splicing affect the resulting translated protein product [8-10]: The first type is exon skipping, in which an exon, also called cassette exon, is spliced out of the transcript together with its flanking introns. The second and third types are the alternative splicing of the 3' splice site and 5' splice site, respectively. Here, two or more splice sites are recognized at one end of the exon. The fourth type is intron retention in which part of an exon is either spliced (like a regular intron) or retained in the mature mRNA transcript. While exon skipping and alternative 3' splice site selection account for most alternative splicing events in higher eukaryotes [11,12], the most prevalent type of alternative splicing in plants, fungi, and protozoa is intron retention [13]. The fifths type is called mutually exclusive splicing and is used for clusters of internal exons that are spliced in a mutually exclusive manner. It is important to note that the term mutually exclusive splicing is only used for these specific clusters of exons. Mutually exclusive splicing demands a specific mechanism for the regulated splicing of exactly one of the exons of such a cluster. Recent analyses have shown that this mechanism might be based on intra-intronic RNA pairings that are conserved at the secondary structure level [14-16]. These alternatively spliced exons must not be mixed up with exons that seem to be spliced in a mutually exclusive manner based on their annotation. This especially accounts for terminal exons that are alternatively spliced in conjunction with the use of alternative promoters or 3'-end processing sites (for a review see for example [17]). The regulation of the splicing of these types need not be at the level of splicing.

To our knowledge, the only study to identify and predict regions *in silico *that might contain mutually exclusive spliced exons used a method of local similarity of genomic regions at the nucleotide level [18]. Assuming that clusters of mutually exclusive exons evolved by one or several rounds of single-exon duplications, given gene locations were self-aligned using a pairwise local alignment algorithm to derive similar regions. Those regions were regarded as candidate regions, and mutually exclusive exons were only predicted by verification through EST and cDNA data. The method itself cannot determine exons including intron splice sites, and is not able to identify mutually exclusive exons whose DNA sequences have diverged considerably. False positive candidates are detected in regions that contain clusters of duplicated genes, and in regions containing pseudo-exons (e.g. exons that are in the process of being lost containing frame-shifts and in-frame stop codons, and missing correct splice sites).

Here, we propose a different approach that is based on the knowledge of creating meaningful transcripts. We presume that most mutually exclusive exons encode the same region of the resulting protein structure. These regions are embedded in the surrounding three-dimensional structure and thus alternative exons must preserve all structurally important contacts between the corresponding local structure elements. A demonstrative example is the alternatively spliced motor domain of the muscle myosin heavy chain in arthropods [19]. In *Drosophila*, four clusters of mutually exclusive spliced exons encode regions of the motor domain, and the variability of creating different transcripts and further fine-tune the motor domain function is even enhanced in the waterflea *Daphnia magna *by four additional clusters. One of the clusters contains exons encoding the so-called relay helix and subsequent relay loop, a structural element that starts at switch-2 embedded in the middle of the motor domain and ends at the connection to the converter domain. This whole relay element converts small conformational changes at the ATP-binding site to large movements of the lever arm [20]. Retaining structural integrity is therefore indispensible for mutually exclusive exons. Of course, parts of the exons might also encode loop regions, but also those parts must at least partly be conserved to retain their general function.

Based on these preconditions we apply the following constrains to our search for mutually exclusive exons: A) Mutually exclusive exons must have about the same length (allowing some length difference for e.g. parts encoding loop regions). B) They must have conserved splice site patterns (e.g. a GT 5' intron splice site cannot be combined with a AC 3' splice site) and the reading frame of the exon must be conserved. C) They must show sequence similarity. These features have been implemented in an extension to the WebScipio software. The application of the algorithm to various genes from several eukaryotes, and to all genes of the X chromosome of *Drosophila melanogaster *is demonstrated.

## Methods

The search algorithm has been implemented as an extension to the WebScipio web application [21]. It is based on the exon-intron gene structure reconstructed by Scipio [22]. The extension is written in the Ruby programming language [23] and fully integrated into WebScipio to facilitate user interaction, and visualization and analysis of the results. WebScipio uses the web framework Ruby on Rails [24]. To make the session storage fast, flexible, and scalable a database backend consisting of Tokyo Cabinet and Tokyo Tyrant [25] is used. To run jobs in background the Rails plug-in Workling in combination with Spawn [26,27] is applied.

### Search algorithm

**Figure 1:**
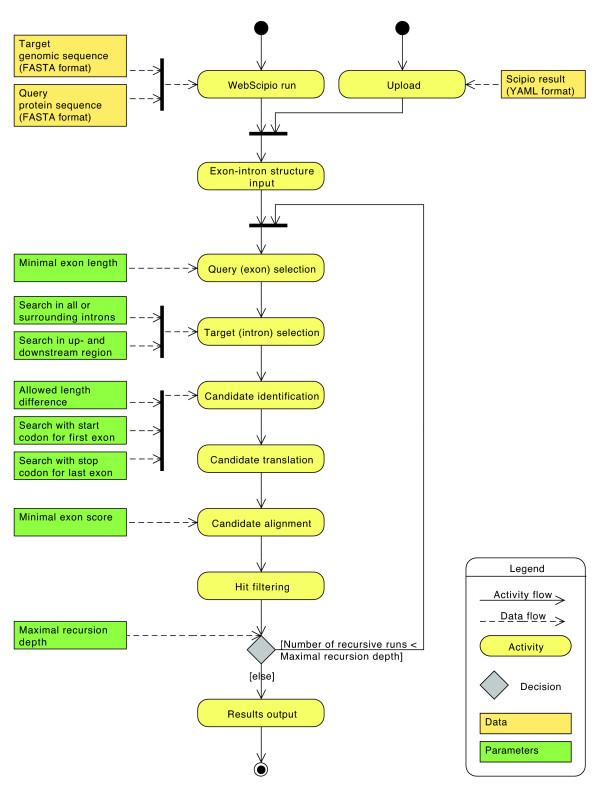
**Activity diagram of the search algorithm**. The activity diagram shows the processing steps of the search algorithm and the influence of the parameters on each step. The run starts with an exon-intron gene structure determined by Scipio. Based on the chosen parameters the exons and corresponding introns are selected and searched for mutually exclusive spliced exon candidates. The candidates are processed and filtered. These steps are repeated in the case of a recursive run. In the end, the algorithm outputs the exon-intron structure including mutually exclusive spliced exons.

The new algorithm divides into several steps, which are executed for each original exon (Figure 1, a detailed activity diagram is available as Additional file 1). It assumes that mutually exclusive spliced exons share the following features: Firstly, mutually exclusive spliced exons have a similar length; secondly, their splice sites and reading frames are conserved; thirdly, they are homologous.

For each internal exon ("original exon") the two surrounding introns (or optionally all introns of the gene) are scanned for exon candidates that have a similar length. These exon candidates must introduce introns with the following splice site pattern: GT---AG, GC---AG, GG---AG, and AT---AC. Firstly, the algorithm looks for the nucleotide pairs AG or AC in the intron sequence, which define start sites of exon candidates and 3' splice sites of the proposed intron. If the intron in front of the original exon starts with GT, GC or GG the algorithm searches for AG, if it starts with an AT the algorithm searches for AC. Secondly, the algorithm looks for the nucleotide pairs GT, GC, GG and AT in the intron sequence, which define ends of exon candidates and 5' splice sites of the proposed intron. If the intron following the original exon ends with AG the algorithm searches for GT, GC and GG, if it ends with AC the algorithm searches for AT. The nucleotide sequences between two possible 3' and 5' splice sites of the scanned intron that have a length similar to the length of the original exon are considered as exon candidates. The maximum length difference between an exon and its candidate can be adjusted by the *allowed length difference *parameter in number of amino acids. The default value of this parameter is 20 aa.

For terminal exons, the algorithm is able to scan the up- and downstream regions of the gene for exon candidates. The first exon of a protein-coding gene has to start with the start codon ATG. Thus, for the first exon, alternative candidates must start with ATG instead of sharing a theoretical splice site pattern with the first exon. The last exon is followed by a stop codon (TAG, TAA, or TGA) and all exon candidates must be followed by a stop codon instead of sharing a splice site pattern with the last exon. The use of the start codon and stop codon instead of the splice sites can be adjusted by the *search with start codon for first exon *and *search with stop codon for last exon *parameters. For example it would be useful to release this restriction in the case where the algorithm searches for alternative exons in a protein fragment. The default of these parameters is to search with a start codon if the first amino acid of the user-provided protein query sequence starts with methionine, and to search with stop codons if the last exon is followed by a stop codon. To reduce the number of candidates it is possible to set the *minimal exon length *parameter. Original exons, which are shorter than this length, are not considered in the candidate search. The default value for this parameter is 15 aa.

The nucleotide sequences of the exon candidates are translated into amino acid sequences using the BioRuby library [28]. The candidates are translated in the same reading frame as the original exon, because their nucleotide sequences appear mutually exclusive in the resulting mRNA and thus share the same reading frame. If the translation results in an in-frame stop codon, the candidate is rejected.

Each candidate sequence is aligned to the original exon sequence. If the alignment score is high, the probability that the two exons are homologous is high as well. The optimal global alignment of the two amino acid sequences is calculated with the Gotoh algorithm, which extends the Needleman-Wunsch algorithm by affine gap costs [29,30]. For this task, the pair_align program of the SeqAn package [31] is used. The gap penalties are set to -10 for initial gaps and -2 for extending gaps. The Blosum62 matrix is used as substitution matrix [32,33]. Because of differences in length and amino acid composition of the clusters of mutually exclusive exons the resulting global alignment scores are not directly comparable. To normalise the alignment scores each score is divided by the score of the alignment of the original exon sequence to itself. This relative score shows the similarity of the two sequences on a scale from zero to one. Candidates, which have a low alignment score, are rejected. The threshold for rejection can be adjusted in percent by the *minimal score for exons *parameter (default: 15%). If candidate regions overlap the highest scoring candidates are retained or, if scores are identical, the longest candidates.

An optional recursive search was implemented to find less similar alternative exons. If this option is selected, the search is repeated with the found alternatively spliced exons as query exons. The number of recursive runs can be adjusted with the *maximal recursion depth *parameter up to three rounds of recursion (default: recursive search disabled).

### WebScipio integration

The WebScipio tool allows reconstructing an exon-intron gene structure based on a protein sequence query. This reconstruction step is the basis for the mutually exclusive spliced exon search. The user can enable the search and adjust several parameters in the Advanced Options section of WebScipio. The search will run subsequently to the gene structure reconstruction step. In addition, the user can enable the search after uploading a previously calculated and downloaded Scipio result.

**Figure 2:**
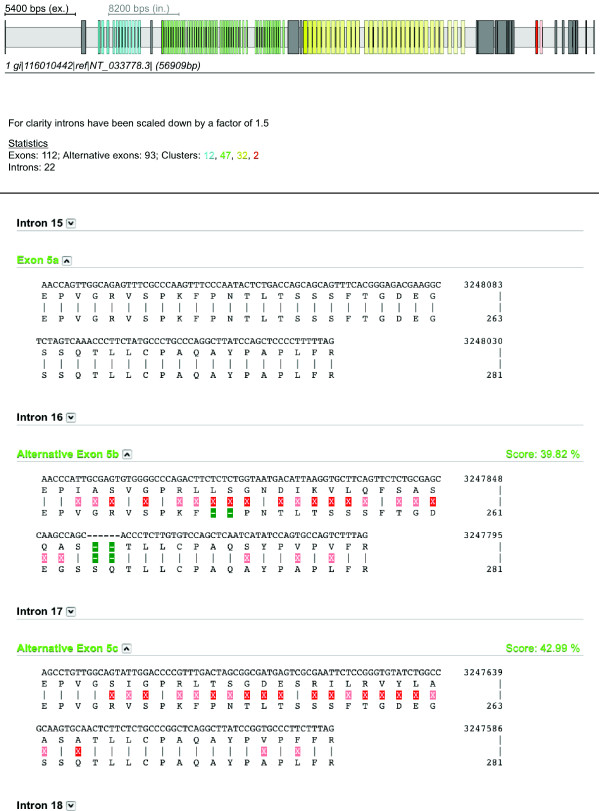
**Gene structure representation and detailed alignment view**. The figure shows the WebScipio gene structure representation of the *Drosophila melanogaster *Dscam gene with mutually exclusive spliced exons and a section of the alignment view including exon 5 and the first two identified alternative exon candidates. The colours in the gene structure figure are the same as the colours of the exon identifiers in the text alignment. The opacity of the colours of each alternative exon corresponds to the alignment score of the alternative exon to the original one. This score is shown in the detailed alignment view next to the exon identifier. For each exon the genomic sequence, its translation, and the translation of the original exon is shown. Identical residues are illustrated as dashes and mismatches as red highlighted crosses. The crosses are highlighted in light red for amino acids, which are chemically similar. Gaps are marked as green hyphens.

The result of the search is displayed in the Result section of the WebScipio interface (Figure 2, top). The standard gene structure picture is extended by the predicted mutually exclusive spliced exons. The alternative exons corresponding to the same original exon constitute a cluster. Exons of a cluster get the same colour. The original exon is dark coloured and the corresponding predicted ones are lighter coloured depending on their similarity with respect to the original exon. In the Statistics section the number of exons in each cluster is shown in colour.

The Alignment view (Figure 2, bottom) offers a detailed analysis at the sequence level. For each alternative exon the genomic sequence, its translation, and the alignment to the original translated exon are shown. The alignment score is given in percent. The alternative exons are also marked in the Genomic DNA result view. In the Coding DNA and Translation result view the user can choose the alternative exons that should build the alternative coding DNAs or protein sequences. The results can be downloaded in several data formats. The YAML file contains all corresponding information and can later be uploaded and used for future analysis [34]. Additionally, the results can be downloaded as General Feature Format (GFF) file [35]. The figures can be downloaded in the Scalable Vector Graphics (SVG) format for further high quality processing [36]. Example searches as well as further descriptions of the search parameters are provided on the help pages of WebScipio.

## Results and Discussion

### Identification of mutually exclusive spliced exons

The search for mutually exclusive spliced exons is based on three criteria: (1) The lengths of the mutually exclusive exons must be very similar, because these exons are supposed to code for the same part in the resulting protein structure, including identical secondary structural elements. (2) To be spliced in a mutually exclusive way, the exons must have similar splice sites and reading frames to be compatible with the previous and following exons. (3) The exons must encode homologous protein sequences, because their inclusion into the protein structure must be compatible with the corresponding local structural environment. The search implemented in WebScipio is based on the availability of the gene structure. Firstly, mutually exclusive exon candidates are searched for using corresponding splice sites to the query exons and restricting the candidate length to similar reading frames (e.g. split codons in the query exon must result in split codons in the candidate exons). Total length difference is less restricted allowing length differences between query and candidate exons at the DNA level in multiples of three for each additional or missing codon. These candidate exons are then filtered and scored based on the Blosum62 matrix. The best scoring, non-overlapping candidates are proposed to be alternative exons to the respective query exon, resulting in a cluster of mutually exclusive exons. With this approach, the absolute necessary constraints at the DNA-level that can be obtained by bioinformatics means are combined with biological information. Based on these criteria several cases can be distinguished: (A) alternative exons found in the surrounding introns of single internal exons should form true clusters of mutually exclusive exons, (B) alternative exons found for terminal exons most probably constitute multiple promoters or multiple poly(A) sites, (C) clusters of several exons in combination, which can be found by searching for candidates for all exons in all introns and up- and downstream regions, most probably represent cases of tandemly arrayed gene duplications or *trans*-spliced genes.

### Example genes with clusters of mutually exclusive exons

**Figure 3:**
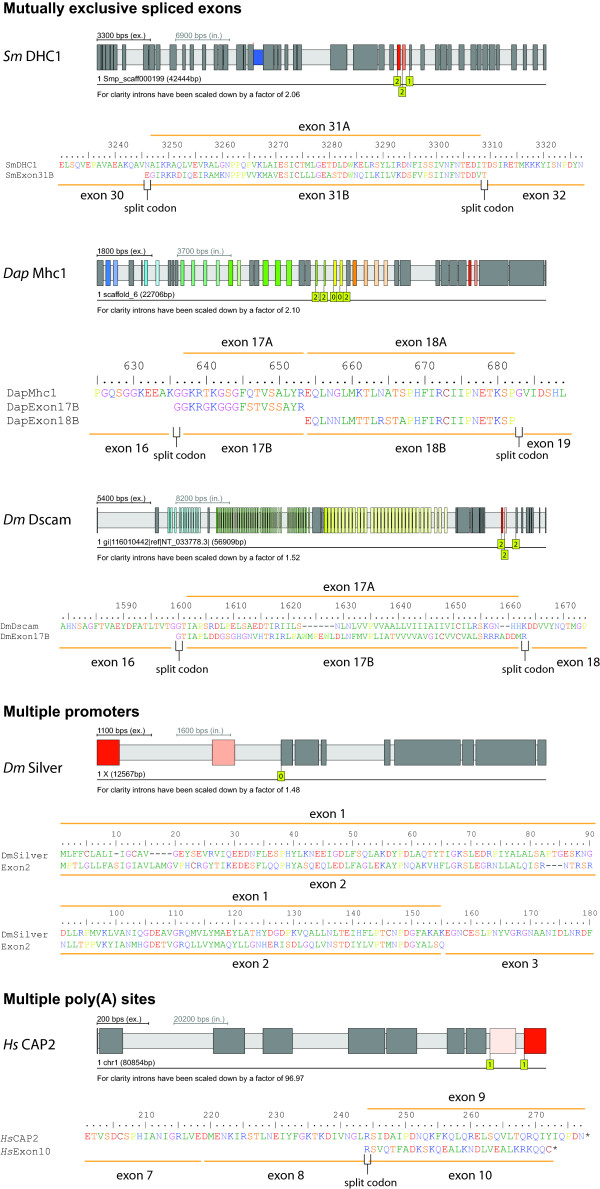
**Example cases of mutually exclusive spliced exons, multiple promoters and multiple poly(A) sites**. The figure illustrates three examples of genes containing mutually exclusive spliced exons, one example containing multiple promoters, and one containing multiple poly(A) sites. Dark grey bars and light grey bars mark exons and introns, respectively. The small blue bar represents an "intron?" that does not have canonical splice sites because an exon is missing in the assembly. Coloured big bars represent mutually exclusive exons found by the new algorithm. The darkest coloured bar is the exon that was included in the query sequence, while the lighter coloured bars represent identified mutually exclusive exons. The higher the similarity between the candidate and the query exon the darker will be the colour of the candidate (100% identity would result in the same colour). Yellow boxes with numbers indicate the reading frame of the corresponding exon.

To test the quality of the new algorithm, several well-known genes with clusters of mutually exclusive exons with different characteristics were analysed (Figure 3). The first test case is the cytoplasmic dynein heavy chain from *Schistosoma mansoni *(*Sm*DHC1). Dynein heavy chains belong to the longest genes in eukaryotes encoding 4000 - 5000 residues and are spread over several dozens of exons. The mutually exclusive exon is clearly identified in the middle of the gene, encoding split codons at the 3'- and 5'-end of the exon. The query exon and the candidate exon have identical lengths and show strong homology. Based on the multiple sequence alignment of more than 2000 DHCs these exons are mutually exclusive and not constitutive or differentially included. The second case represents the muscle myosin heavy chain gene from the waterflea *Daphnia magna *[19]. The arthropod muscle myosin heavy chain genes contain several clusters of mutually exclusive exons to fine tune the mechanochemical characteristics of the motor domain that are needed to accomplish the different tasks in the various muscle types [37]. The *Dap*Mhc1 is an example with nine clusters of mutually exclusive exons of which several are adjacent and not interrupted by constitutive exons. The new algorithm found all mutually exclusive exons that have manually been identified previously [19]. The two example alignments show that the new algorithm is able to correctly identify even short exons with limited complexity, and subsequent clusters of mutually exclusive exons encoded in different reading frames. The third example shows the prediction of the mutually exclusive exons in Dscam (Down syndrome cell adhesion molecule) from *Drosophila melanogaster*, which is known to encode the largest set of mutually exclusive exons of any gene analysed so far [38,39]. The potentially 95 mutually exclusive exons of the Dscam gene are organized into four clusters that are separated by constitutive exons. The exon 4, 6, 9, and 17 clusters are supposed to contain 12, 48, 33, and 2 exons, respectively [39]. In the publicly available *Drosophila melanogaster *reference genome sequence (chromosome assembly version 4.1 as provided by Flybase [40,41]) mutually exclusive exons were searched using a gene translation containing the first exons of each of the clusters as query sequence. If clusters contain that many exons as are found in the Dscam genes it might be possible that the exon, that has been included in the query sequence, is the most divergent of the exons of the cluster. Therefore, a parameter to the search algorithm that enforces recursive searches in all introns with the newly identified exon candidates was introduced. Exons that might not be identified in the first round might then be found in the second, third, or later round. Of course, the recursive depth should not be too large to avoid the inclusion of false positive exons because of the decreasing stringency of the query exons. Including every first exon of the Dscam mutually exclusive exon clusters in the query sequence, all twelve exons of the exon 4 cluster were identified, both exons of the exon 17 cluster, and 46 and 32 exons for the exon 6 and exon 9 cluster, respectively (Figure 3, Table 1). Increasing the recursive depth to one also revealed exon 6.11, which is the most divergent exon of the cluster, and which has not been detected in transcriptome studies yet [42-44]. Exon 6.47 was not identified because the intron before exon 6.47 does not have an "AG" at the 3'-end and is therefore not compatible with the "GT" at the 5'-end of the intron succeeding exon 5. The supposed 5'-end sequence of exon 6.47 is different to the published sequence [39] but is supported by many genomic DNA reads available from GenBank (a genomic DNA read identical to the published sequence was not found). Exon 9.13 was also not identified because it contains a frame shift in the *Drosophila *reference genome assembly, supported by many genomic DNA reads. Therefore, the translations of the predicted transcripts containing exon 9.13 all stop shortly behind this frame shift (e.g. NM_001043054.1, NM_001043034.1, and NM_001043065.1). However, both exon 6.47 and exon 9.13 were identified in many transcripts [42-44]. Thus, either the genome assembly based on the many genomic DNA reads is wrong, which is unlikely, or the many EST/cDNA-reads are wrong, which is also unlikely, or the genomic DNA has been obtained from a different strain than the one that has been used in the transcriptome studies. WebScipio is, however, not able to identify mutually exclusive exons if those do not correspond to the exon length (e.g. frame shifts will result in other reading frames and exon lengths) and corresponding splice site restrictions. The strength of the new algorithm is illustrated at the exon 17 cluster that encodes two highly divergent but mutually exclusive spliced exons (Figure 3). When applying the search for mutually exclusive exons in the Dscam gene against the published genomic sequence (NCBI accession number AF260530 [39]) all proposed 95 mutually exclusive exons were identified (Table 1). Less mutually exclusive exons in the search against the *Drosophila melanogaster *reference genome sequence compared to the search against the published sequence are therefore not due to problems with the search algorithm.

**Table 1:** Mutually exclusive exons in the *Drosophila *species Dscam genes

exon	Dm	AF260530	**Dse**^**a**^	Dy	Der	Da	Dp	**Drp**^**a**^	Dw	Dmo	Dv	Dg
4	12	12	12	12	12	12	12	10	12	12	12	12
6^b^	46/47	47/48	46^c^	39/40	44	47	49	49	48/49	50	52	53
9	32	33	29	32	33	33	32	29	29	32	32	32
17	2	2	2	2	2	2	2	2	2	2	2	2
total	92/93	94/95	90	85/86	91	94	95	90	91/92	96	98	99

[16,46]	95	95	95	87	94	93	94	95	95	95	98	94
[15]	95	95		88		93	94			95	98	

Dm = *Drosophila melanogaster*; Dse = *Drosophila sechellia Rob3c*; Dy = *Drosophila yakuba Tai18E2*; Der = *Drosophila erecta TSC#14021-0224.01*; Da = *Drosophila ananassae TSC#14024-0371.13*; Dp = *Drosophila pseudoobscura MV2-25*; Drp = *Drosophila persimilis MSH-3*; Dw = *Drosophila willistoni TSC#14030-0811.24*;

Dmo = *Drosophila mojavensis TSC#15081-1352.22*; Dv = *Drosophila virilis TSC#15010-1051.87*; Dg = *Drosophila grimshawi TSC#15287-2541.00*.

^a ^Genomes are fragmented and contain gaps in the Dscam genes.

^b ^The first number corresponds to a search with standard parameters, the second to searches with one round of recursion.

^c ^One of the exons is a pseudo-exon because it misses the last forth of the exon because of an in-frame stop codon.

In addition, mutually exclusive exons in the Dscam genes of the other sequenced *Drosophila *species were searched ([45]; Table 1). Here, all mutually exclusive exons were found immediately, and only three further exons were identified by a second recursive round of exon search. As found for the *Drosophila melanogaster *gene, WebScipio identified sometimes more sometimes less exons compared to the published analyses [15,16,46]. However, the WebScipio searches were performed against the official reference genome assemblies, while the published analyses were based on manually performed genomic clone assemblies of the Dscam gene regions. Therefore, the differences in exon numbers do not result from shortcomings of the search algorithm, but from differences in the assembly of the reference genome data and the manually assembled genomic regions.

### Example genes encoding 5'- and 3'-terminal exons with features of mutually exclusive spliced exons

Terminal exons are often not selected at the level of splicing. Instead, initial (5'-terminal) exons are most probably selected at the level of transcription that starts at different promoters. Terminal exons (or better alternative exons encoding for the terminal stop codon) might either be spliced as differentially included exons, like in the case of the *Drosophila *muscle myosin heavy chain gene [19], or as multiple poly(A) sites. Nevertheless, these terminal exons might contain an important structural part of the encoded protein and thus often have similar length and show sequence similarity. Figure 3 shows two examples of genes that contain 5'- and 3'-terminal exons sharing the described features of mutually exclusive exons, but are spliced as multiple promoters or multiple poly(A) sites. The silver protein of *Drosophila melanogaster *illustrates a case where two initial exons, which are transcribed/spliced as multiple promoters, share the features of mutually exclusive exons. The capping protein beta (Capβ) from *Homo sapiens *represents a case where homologous 3'-terminal exons containing multiple poly(A) sites are found. The detection of these cases can be suppressed by disabling the search for mutually exclusive exons for 5'- and 3'-terminal exons. By default, WebScipio enables the search for homologous exons for all exons, because it is not known whether the user is searching with a complete, partial or fragmented query sequence. In the case of partial and fragmented sequences the search would provide significant results. Also, genes sometimes contain untranslated 5'- and/or 3'-terminal exons whereby the first translated exon could well be part of a cluster of mutually exclusive spliced exons. In addition, alternative terminal exons by themselves might provide interesting perspectives to the corresponding genes independently of whether they are mutually exclusively spliced or not. WebScipio cannot distinguish between the described cases and thus the user has to be careful when alternative terminal exons are proposed.

### Detection of *trans*-spliced genes and arrays of tandem gene duplications

**Figure 4:**
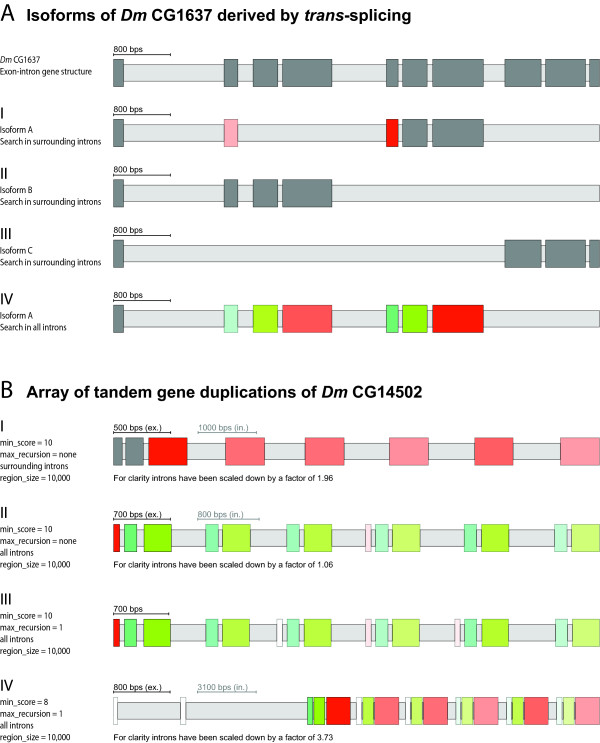
**Examples of a *trans*-spliced gene and an array of tandem gene duplications**. A) Schematic representation of the *trans*-spliced *Drosophila melanogaster *CG1637 gene. The three annotated isoforms A-C are shown consisting of the common 3'-terminal start exon and different groups of alternative exons. If only isoform A were known a potentially mutually exclusive exon would have been found by a search for candidates in surrounding introns (case I). However, a search for candidates of all exons in all introns reveals the two groups of homologous exons that are *trans*-spliced in isoform A and B (case IV). Isoform C also encodes a cluster of *trans*-spliced exons whose sequence is homologous to that of isoform A/B. However, the exonic sequence is interrupted at different intron positions (case III). Note, that the gene structure annotated by Flybase (shown here) is different to the published one ([46], supplementary Figure 3). B) Gene duplications of the *Drosophila melanogaster *CG14502 gene. The figure shows the tandem arrangement of the duplicated genes of the *Drosophila melanogaster *CG14502 gene as found by WebScipio. The parameters *minimal score for exons*, *maximal recursion depth*, *search in all introns *and *region size *were adjusted for each search. With less restrict parameters less similar exons are found.

The *trans*-splicing of separate pre-mRNAs involving coding exons to reveal a joined transcript is a relatively uncommon event [47]. In general, *trans*-spliced genes in *Drosophila melanogaster *can be distinguished into those with multiple first exons or multiple 3'-terminal exons, or those with very large introns. Many of the *trans*-spliced genes contain variable single terminal exons (e.g. *mod(mdg4) *[48,49] or *lola *[50]) or alternative terminal exon groups (e.g. CG42235 [47]). When searching for mutually exclusive spliced exons based on one of the annotated isoforms of a *trans*-spliced gene potentially alternative exons of internal exons might be identified. An example of the *trans*-spliced *Drosophila melanogaster *gene CG1637 is shown in Figure 4A. Three isoforms of the CG1637 gene exist (Isoform A, B, and C) that result in transcripts of a common 5' exon spliced to isoform-specific sets of three 3' exons. The sequences of the isoform-specific sets are homologous although the intron positions are different between the isoform A/B exons and the isoform C exons. When searching with the isoform A exons for mutually exclusive exons in surrounding introns the homologous exon of isoform B is found for the first of the three isoform A-specific exons (Figure 4A-I). When only searching in surrounding introns (search in up- and downstream regions disabled) further exons are not found for isoform B (homologous exons would only exist in the downstream region, Figure 4A-II) and for isoform C (the introns are at different positions so that the similar-length condition does not apply anymore, Figure 4A-III). Thus, if only isoform A were known a mutually exclusive exon would have been proposed. To avoid the mis-annotation of exons of *trans-*spliced clusters a parameter was introduced that allows searching for candidate exons not only in the neighbouring introns but also in all introns. In Figure 4A-IV the exons of isoform B were identified by searching with the exons of isoform A in all introns revealing the *trans*-spliced nature of the cluster.

If searching in up- and downstream regions for alternatively spliced exons, it is possible that candidate exons belong to gene duplicates (Figure 4B). In this case, the WebScipio option to search for candidates in all introns including up- and downstream regions and not only in surrounding introns helps identifying exons of gene duplications. In many cases, gene duplications result in genes arranged in tandem. Those gene duplicates often share the complete gene structure meaning that for every exon there is a corresponding exon in the duplicated gene. Figure 4B illustrates this behaviour and provides means by which users can judge between a true cluster of mutually exclusive exons belonging to one gene and a set of duplicated genes. If the search for candidate exons is only allowed in surrounding introns, a set of six homologous exons is found for the *Drosophila melanogaster *gene CG14502 (Figure 4B, I). Performing the search in all introns results in five homologous exons also for the second exon of the CG14502 gene, and shows one homologous exon for exon 1 (Figure 4B, II). The first exons of the genes seem to be very divergent. Allowing one additional recursive round of candidate search reveals the first exons for two additional gene homologs (Figure 4B, III). In addition lowering the score reveals the exon 1 candidates of the remaining two gene homologs, although two further regions with very low homology to exon 1 appear in the upstream region of the CG14502 gene (Figure 4B, IV). This example illustrates the use of the search parameters so that gene duplications can be identified. Gene duplicates that are not arranged in tandem but are distributed in the genome do not provide problems in evaluating exon candidates, because the search is restricted to a certain size of the up- and downstream regions. If needed, these gene duplicates can be identified with WebScipio using the general *multiple results *option.

### Application of the search algorithm for mutually exclusive exons to genome scale data

The described search algorithm identifies three types of exons as described above: (A) mutually exclusive exons, (B) terminal exons that are spliced as multiple promoters or multiple poly(A) sites but share similar length, reading frame, and sequence homology, and (C) exons with the characteristics of mutually exclusive exons that are actually part of tandemly arrayed gene duplicates or groups of alternative exons in *trans*-spliced genes. Type B and type C exons are false positives, when looking for mutually exclusive exons. In addition, false positive exons are those exons that show all characteristics of type A exons but are constitutively or differentially included spliced. False negatives exons, which are not identified by WebScipio, are those mutually exclusive exons that do not have similar length and sequence homology. To quantify the amount of each of these exon types we searched the complete X chromosome of the fruit fly *Drosophila melanogaster *for mutually exclusive spliced exons with WebScipio and compared the results to the Flybase annotation.

Protein sequences for the search were obtained from the Flybase annotation (version 5.27) and mapped to the genomic sequence of the X chromosome using Scipio. 2,967 transcripts containing more than one exon were derived from 1,705 genes. For each exon mutually exclusive alternative splice variants have been searched for in the surrounding introns. The search parameters were set to 20 amino acids for the *allowed length difference*, to 15% for the *minimal score for exons*, and to 15 amino acids for the *minimal exon length*. We did not search for alternative exons in up- and downstream regions of genes, and we did not apply the recursive search, which means the repeated search for further alternative exons with the newly identified exons that we demonstrated for Dscam (see above). Three genes (lethal (1) G0193, CG1637, and CG42249), in which mutually exclusive exons were found, were excluded from the analysis, because the respective exons are spliced in a mutually exclusive manner in groups of two, three, and four exons, instead of single exons within a cluster. Those genes are probably *trans*-spliced (for an example see Figure 4A).

### Search for non-mutually exclusive exons sharing similar length, same reading frame, and sequence homology

It could well be possible that internal exons with similar length, same reading frame, and showing sequence homology are not mutually exclusive spliced exons, but constitutive exons or exons spliced by one of the other types of alternative splicing. To get a statistically relevant number of these types of exons we collected all genes of the *Drosophila melanogaster *X chromosome containing at least two exons based on the Flybase annotation version 5.27. The transcripts of each gene were analysed independently because alternative splicing produces different exon neighbours. Thus exons are counted for each transcript (not each gene) even if the transcripts have the same start and end points in the genomic sequence. In total, the 2,967 transcripts of the *Drosophila melanogaster *X chromosome include 16,180 exons. All neighbouring exons were compared with respect to having similar length (*allowed length difference *20 aa), sharing high similarity (*minimal score for exons *15%), coding for at least fifteen amino acids (*minimal exon length *15 aa), and encoding the same reading frame. The results are summarized in Table 2 (for detailed information see Additional file 2). Only 0.56% of the non-mutually exclusive exons (90 out of 16180) share the features of mutually exclusive exons. These exons are located in only six genes out of 1705 (0.35%). In one of the six genes (*Ciboulot*) the two homologous exons are terminal exons and would represent a case of multiple poly(A) sites if alternatively spliced. This analysis shows that the chance that the exons predicted by WebScipio as mutually exclusive exons will later (e.g. after obtaining cDNAs) be reannotated as constitutive or differentially included exons, is very low.

**Table 2:** Search for exons annotated as constitutively spliced or differentially included sharing similar length, same reading frame and sequence homology in the *Drosophila melanogaster *X chromosome

	Total	**Hits**^**a**^	Percentage
Exons	16180	90	0.56%
Transcripts	2967	20	0.67%
Genes	1705	6	0.35%

^a ^Exons (or transcripts/genes containing exons) which share similar length, same reading frame and sequence homology

### Search for mutually exclusive spliced exons in the *Drosophila melanogaster *X chromosome

Some categories have to be defined to separate true (annotated) mutually exclusive spliced exons from predicted ones and false positives and false negatives. As real mutually exclusive exons we regard those with the following criteria: An exon is part of a cluster of mutually exclusive spliced exons if each transcript of the gene contains exactly one exon of the cluster (not none or more than one), the cluster contains at least two exons, the exons of the cluster are neighbouring exons, and the cluster is surrounded by further exons. The latter criterion distinguishes the mutually exclusive spliced exons from clusters of initial exons (5'-terminal exons) and 3'-terminal exons that are spliced in a mutually exclusive manner and share sequence similarity, similar length, and splice site conservation. In contrast to real mutually exclusive spliced exons the exons of these clusters appear mutually exclusive in the transcripts but their transcription and splicing is regulated in a different way. These clusters are therefore regarded as types of multiple promoters and types of multiple poly(A) sites, and are false positives. Other types of false positives are those exons that are predicted by WebScipio but overlap with already annotated exons and do not match exactly the positions of these exons. False negatives are those exons that do not meet the preconditions of similar length and sequence homology. However, if those exons are mutually exclusive spliced they must have conserved splice sites and reading frames.

**Figure 5:**
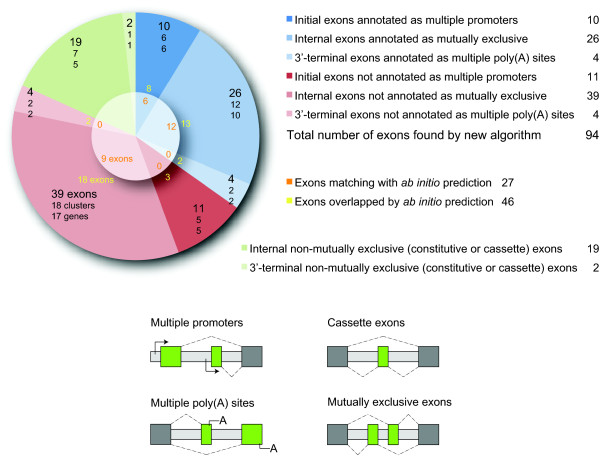
**Exons located on the *Drosophila melanogaster *X chromosome sharing similar length, same splice sites and reading frames, and sequence similarity**. The pie chart shows the total number of exons of the *Drosohpila melanogaster *X chromosome, which share the features used by the new search algorithm. The blue and red slices represent the number of exons found by the new algorithm compared to existing annotations and to the *ab initio *prediction by AUGUSTUS, shown in the middle. The blue part illustrates the exons already annotated by Flybase, in contrast to the exons in clusters additionally predicted by WebScipio in red. The pie is divided in slices for initial, internal, and 3'-terminal exons. In addition to the number of exons, the chart indicates the number of clusters and genes, in which these exons were found. The orange numbers in the middle part of the pie indicate how many of the respective exons are found and reconstructed with correct exon borders by the *ab initio *prediction with AUGUSTUS, while the yellow numbers reveal the number of exons to which exons predicted by AUGUSTUS at least overlap. The green slices indicate constitutive exons, which share the features of mutually exclusive exons. These are the same exons, clusters, and genes as listed in Table 2 and Additional file 2. At the bottom, the figure illustrates the different types of alternatively spliced exons (multiple promoters, multiple poly(A) sites, mutually exclusive exons) in comparison with the cassette exon type.

In total, 94 exons of similar length, same splice sites and reading frames, and sequence homology have been identified by WebScipio, of which 65 are potentially in clusters of mutually exclusive exons, 21 in clusters of multiple promoters, and 8 in clusters of multiple poly(A) sites (Figure 5). Of the 65 exons predicted to belong to internal clusters of mutually exclusive spliced exons, 26 exons are already annotated in Flybase. 39 exons are predictions by WebScipio that have not been described before. These 39 exons are distributed into 18 clusters that belong to 17 genes. Thus, there are several clusters with more than two alternative exons, and one gene with two clusters. If the Flybase based annotation is assumed to represent true mutually exclusive exons, the chart represents the specificity of our method. The 26 already known mutually exons divided by 65 predicted exons result in 40% specificity. However, the value for the specificity is misleading because it depends on the "known" mutually exclusive exons. We expect that many of the additional exons predicted by WebScipio will be experimentally confirmed in the future and thus will become "known" mutually exclusive exons. The true specificity will therefore be much higher than the value of 40% suggests. To analyse whether the additional exons predicted by WebScipio contain general features of exons (for example a higher GC content than the surrounding region), the found exons were compared to those of an *ab initio *prediction performed by AUGUSTUS [51] (Figure 5). In many cases the WebScipio predictions are supported by the *ab initio *prediction, which is based on the genomic sequence alone. The AUGUSTUS prediction matches 27 of the 94 exons with exact exon borders (Figure 5, orange numbers) and overlaps with 46 of them (Figure 5, yellow numbers).

The results show that about 70% of all predicted exons (65 out of 94) comprise clusters of internal mutually exclusive exons. The false positive prediction of 5'- and 3'-terminal exons as mutually exclusive exons, which comprise the remaining 30% of predicted exons, could even be suppressed by a WebScipio option. We can also conclude that WebScipio correctly identifies all but one (see following section) of the annotated mutually exclusive exons. This suggests that most of the WebScipio predictions of new mutually exclusive exon candidates will also be real mutually exclusive exons. This is supported by the *ab initio *exon prediction by AUGUSTUS that showed exon probability for about 50% of the newly predicted exons, which is comparable to the *ab initio *prediction of the already annotated exons. However, we cannot completely exclude the possibility that some of the newly predicted exons might in truth be constitutive or differentially included exons (see previous section).

**Figure 6:**
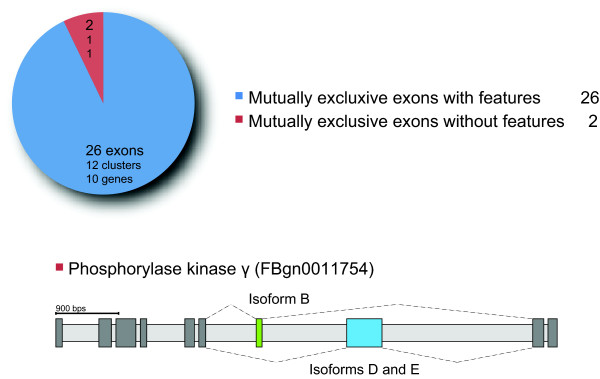
**Mutually exclusive exons in genes of the *Drosophila melanogaster *X chromosome**. The figure illustrates how many of the mutually exclusive exons, which were annotated based on Flybase transcripts, share the following features: high sequence similarity, similar length, same reading frame, and a minimal exon length (15 residues). The blue slice indicates exons characterised by these features and found by the new algorithm. The red slice indicates exons not sharing these features. At the bottom, the figure shows the exon-intron structure of the Phosphorylase kinase γ gene, which includes the only cluster of mutually exclusive exons that was not found by the new algorithm.

False negatives would be those mutually exclusive spliced exons that do not share a similar length and sequence similarity. To figure out how often clusters of mutually exclusive exons with such characteristics exist in comparison to mutually exclusive exons with similar length and sequence similarity, all internal clusters of exons on the X chromosome that were annotated as mutually exclusive based on Flybase transcripts were manually analysed (Figure 6). Of the annotated genes only the Phosphorylase kinase γ gene contains two mutually exclusive spliced exons that do not have similar length and sequence (Figure 6, bottom). If the Flybase annotation is assumed as true, the chart in Figure 6 represents the sensitivity of the algorithm. 26 predicted mutually exclusive spliced exons divided by 28 annotated exons results in 93% sensitivity for internal exons. These data likely indicate that not many mutually exclusive spliced exons will be missed given the constraints of similar length and sequence similarity as implemented in WebScipio.

**Figure 7:**
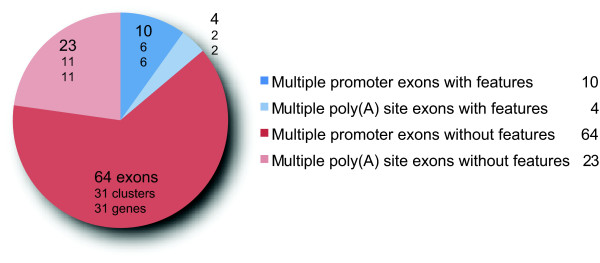
**Exons belonging to clusters of multiple promoters and multiple poly(A) sites in the *Drosophila melanogaster *X chromosome**. The figure shows the number of multiple promoter exons and multiple poly(A) sites exons based on the Flybase annotation and illustrates how many of these exons share the following features: high sequence similarity, similar length, same reading frame, and a minimal exon length (15 residues). Blue slices indicate exons characterised by these features, and red slices indicate exons not sharing these features.

Mutually exclusive exons predicted for 5'- and 3'-terminal exons were regarded as false positives because these rather present cases of multiple promoters, multiple poly(A) sites, and differentially included exons. However, additional untranslated terminal exons might exist that were not analysed here, and in those cases the exons, based on the translation predicted as terminal, become internal and thus true mutually exclusive exons. For comparison all terminal exons annotated as transcribed or spliced in a mutually exclusive manner have been analysed (Figure 7). Of the 101 terminal exons only 14 terminal exons share the features of mutually exclusive spliced exons. A reason for the sequence and length variability of terminal exons is that the N- and C-termini of proteins are not as restricted in their structure as internal parts. Thus, the number of false positives predicted by WebScipio is rather low.

### Future developments and applications

Due to the precondition that mutually exclusive exons encode the same part of the protein product, we also want to include the comparison of the prediction of secondary structural elements for the query and the candidate exons as an additional scoring, analysis, and validation parameter. Also, other substitution matrices might be offered for the scoring of the aligned query and candidate exons. Scipio and WebScipio have been shown to be suitable for the prediction of genes in cross-species searches [21,22]. Of course, both approaches can be combined and users can search, for example, with a human protein query sequence in other mammals to identify homologous genes and simultaneously predict mutually exclusive exons in the target sequence. Because the search for mutually exclusive exons relies on the translation of the exons as found in the genomic DNA, it does not depend on the initial query sequence but on the quality of the exons identified in the cross-species search. Another application would be to search for mutually exclusive spliced genes in the complete genomes of sequenced eukaryotes.

## Conclusions

The extension of WebScipio to search for mutually exclusive exons is based on the precondition that these exons encode regions of the same structural part of the protein product. This precondition provides restrictions to the search for candidate exons concerning their length, splice site conservation and reading frame preservation, and overall homology. The implemented algorithm has been shown to identify all known mutually exclusive spliced exons in many example genes from various species, like the muscle myosin heavy chain gene of *Daphnia pulex *or the Dscam gene of *Drosophila melanogaster*. The search for homologs of terminal exons might, however, result in the prediction of multiple promoters, multiple poly(A) sites, groups of *trans*-spliced exons, or tandemly arrayed gene duplicates, and can therefore optionally be disabled. To quantify the quality of WebScipio to correctly predict already annotated mutually exclusive exons and to predict so far unrecognized exon candidates, an analysis of the whole X chromosome of *Drosophila melanogaster *has been performed. All but two of the 28 annotated mutually exclusive exons were found by WebScipio. In addition, WebScipio predicts 39 new mutually exclusive exon candidates of which about 50% are supported by an *ab initio *exon prediction by AUGUSTUS. In conclusion, WebScipio should be able to identify mutually exclusive spliced exons in any query sequence from any species with a very high probability.

## Abbreviations

DHC: Dynein heavy chain; Dscam: Down Syndrome Cell Adhesion Molecule; GFF: General Feature Format; Mhc: Myosin heavy chain; SVG: Scalable Vector Graphics; YAML: YAML ain't markup language

## Authors' contributions

HP, FO, and MK set the requirements for the system. HP and KH wrote the software. FO assisted in and supervised the implementation of the software. BH implemented improvements to the software, and KH committed the final version. KH performed the whole chromosome search and evaluation. HP, KH, BH, and MK performed extensive testing. KH and MK wrote the manuscript. All authors read and approved the final version of the manuscript.

## Supplementary Material

Additional file 1 **Detailed activity diagram**. The detailed activity diagram shows each step of the search algorithm including points of decision and loops. Click here for file

Additional file 2 **Search for non-mutually exclusive exons sharing similar length, same reading frame and sequence homology**. The file provides detailed information of the found genes and their gene structures. Click here for file
