# Supplementary figures and images for: Predicting mutually exclusive spliced exons based on exon length, splice site and reading frame conservation, and exon sequence homology
Source: BMC Bioinformatics. 2011 Jun 30;12:270. doi: 10.1186/1471-2105-12-270 (PMC3228551; doi:10.1186/1471-2105-12-270)

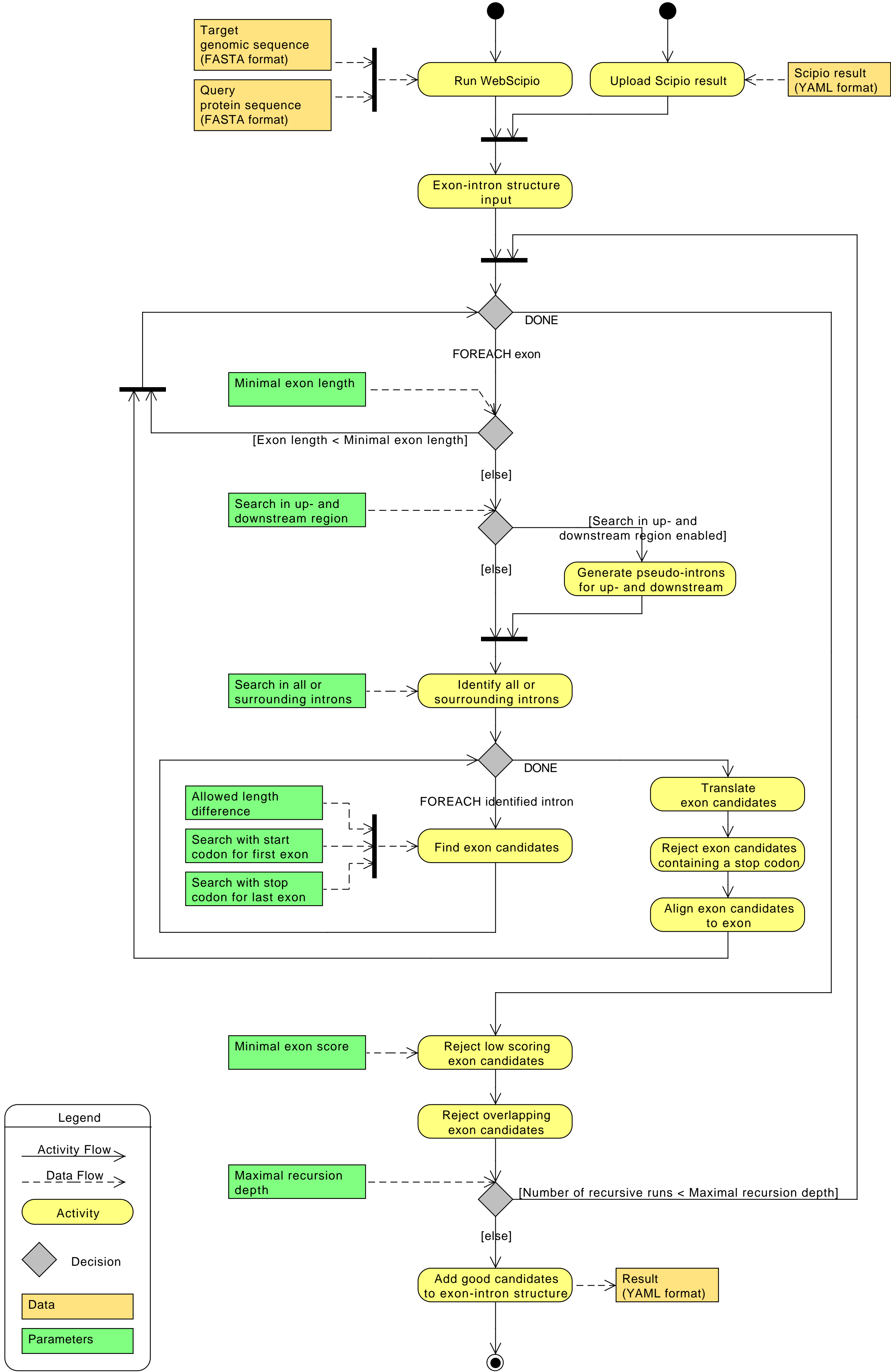

Supplement: Additional file 1 — Detailed activity diagram. The detailed activity diagram shows each step of the search algorithm including points of decision and loops. [file 1471-2105-12-270-S1.PDF]
