# Supplementary material for: Predicting mutually exclusive spliced exons based on exon length, splice site and reading frame conservation, and exon sequence homology
Source: BMC Bioinformatics. 2011 Jun 30;12:270. doi: 10.1186/1471-2105-12-270 (PMC3228551; doi:10.1186/1471-2105-12-270)

Search for non-mutually exclusive exons sharing similar length, same reading frame and sequence homology

All genes of the *Drosophila melanogaster* X chromosome containing at least two exons were collected based on the FlyBase annotation version 5.27. In all transcripts of the 1,705 genes neighboring exons were identified, which code for at least fifteen amino acids (minimal length 15aa), share high similarity (score >= 15%), have similar length (length difference <= 20aa) and are in the same reading frame. The following table shows the six genes, which contain 21 of those exons distributed in 8 clusters. The figures show the detailed gene structure of those genes. The arrows indicate the length differences and scores of the similar exons.

Gene Name	Flybase ID	Exon hits per gene	Clusters per gene	Score %	Length difference [aa]
C901	FBgn0021742	2	1	51 – 59	11
CG15570	FBgn0029697	5	2	32 – 38	2 – 3
Ciboulot	FBgn0026084	2	1	30 – 35	8
Megalin	FBgn0261260	2	1	22	6
Tenascin accessory	FBgn0259240	3	1	27 – 36	5 – 8
Terribly reduced optic lobes	FBgn0261451	7	2	17 – 30	3 – 6
All 6 genes		21	8	17 – 59	2 – 11

Gene: C901, FBgn0021742
Polypeptide: C901-PA , FBpp0073256

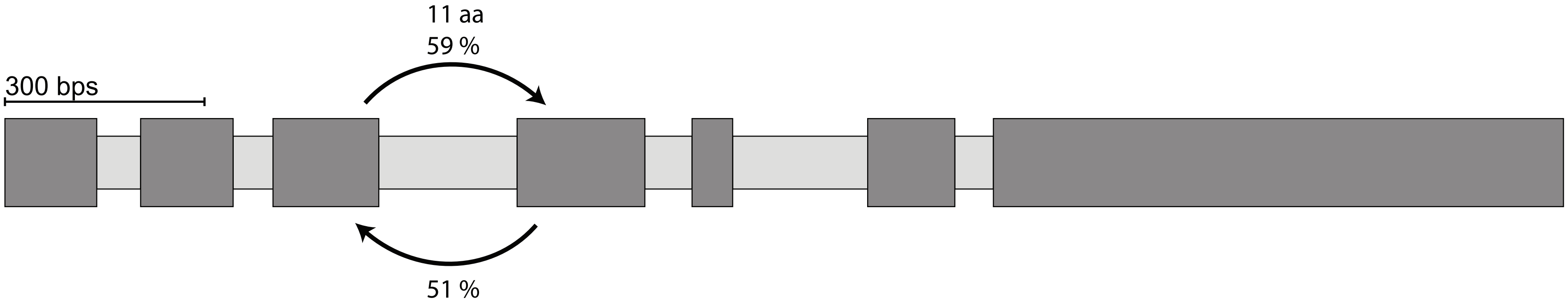

Gene: CG15570, FBgn0029697
Polypeptide: CG15570-PA, FBpp0070613

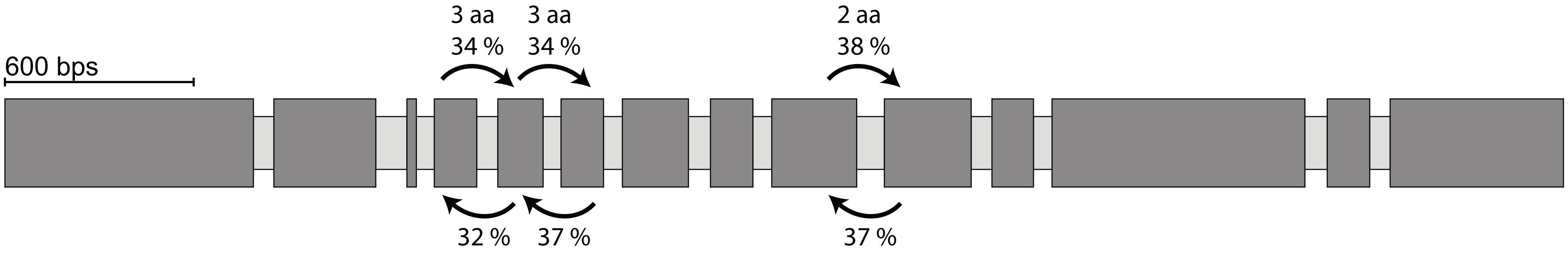

Gene: Ciboulot, FBgn0026084
Polypeptide: cib-PA, FBpp0070607

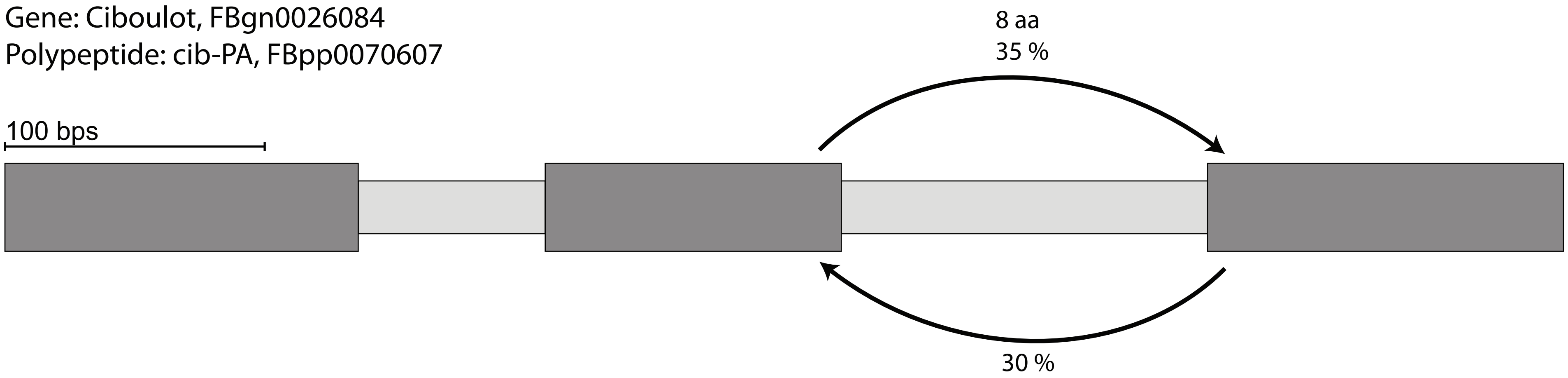

Gene: Megalin, FBgn0261260
Polypeptide: Megalin-PA, FBpp0291363

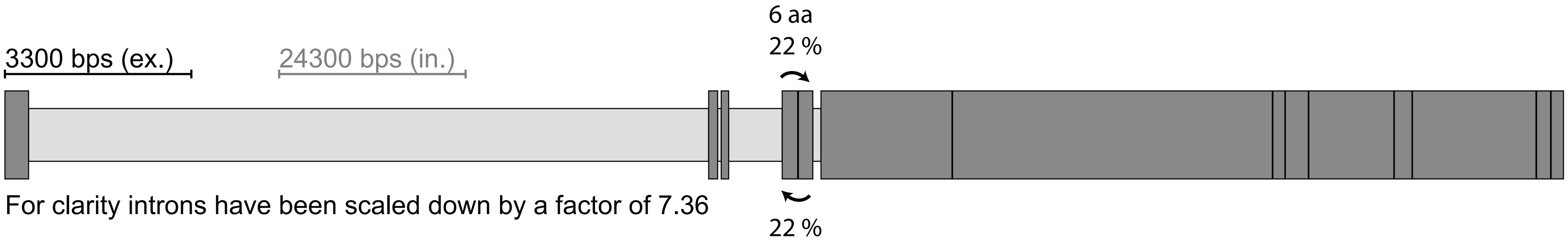

Gene: Tenascin accessory, FBgn0259240
Polypeptide: Ten-a-PD, FBpp0289136

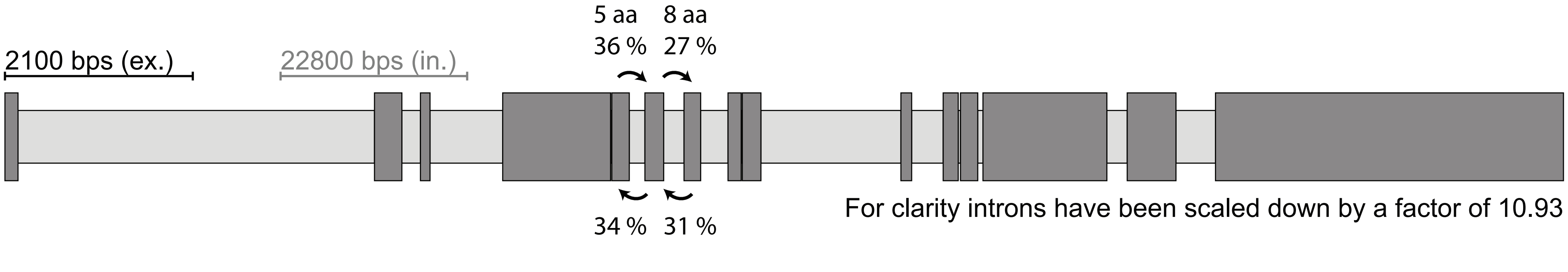

Gene: Terribly reduced optic lobes, FBgn0261451
Polypeptide: trol-PD, FBpp0070440

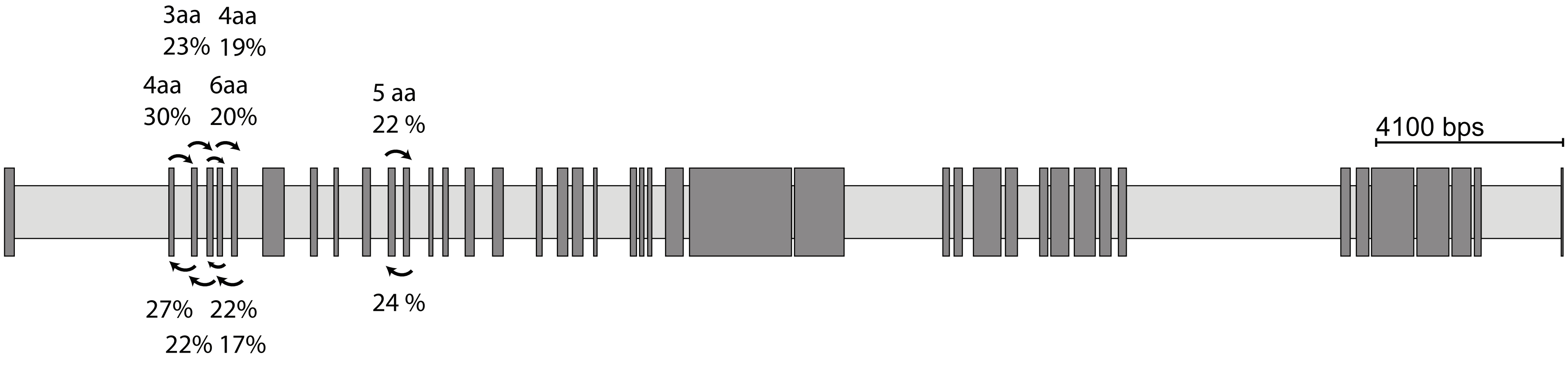

Supplement: Additional file 2 — Search for non-mutually exclusive exons sharing similar length, same reading frame and sequence homology. The file provides detailed information of the found genes and their gene structures. [file 1471-2105-12-270-S2.PDF]
